# Impact of dehydroepiandrosterone sulfate and free androgen index on pregnancy and neonatal outcomes in PCOS patients

**DOI:** 10.1186/s12958-024-01212-y

**Published:** 2024-04-16

**Authors:** Wen Zhao, Zeting Li, Bing Cai, Canquan Zhou, Qingyun Mai

**Affiliations:** 1 Reproductive Medicine Center, The First Affiliated Hospital, Sun Yat-sen University, Guangzhou, Guangdong China; 2 Key Laboratory of Reproductive Medicine of Guangdong Province, Guangzhou, People’s Republic of China; 3 Guangdong Provincial Clinical Research Center for obstetrical and gynecological diseases, Guangdong, China; 4 Department of Endocrinology, The Seventh Affiliated Hospital, Sun Yat-sen University, Shenzhen, China

**Keywords:** Polycystic ovary syndrome (PCOS), Androgens, Cumulative pregnancy rates (CPR), Cumulative live birth rates (CLBR), Free androgen index (FAI), Dehydroepiandrosterone sulfate (DHEAS)

## Abstract

**Background:**

Polycystic ovary syndrome (PCOS) is a prevalent endocrine disorder associated with infertility and pregnancy complications. The pathogenesis of PCOS and its impact on reproductive function may be influenced by the source of androgens, including testosterone, free androgen, dehydroepiandrosterone sulfate (DHEAS). However, the differential effects of these androgen on pregnancy and neonatal outcomes and the cut-off value of East Asian population with PCOS remain unclear.

**Methods:**

A retrospective cohort study was conducted at the Reproductive Medicine Center of the First Affiliated Hospital of Sun Yat-sen University from January 2015 to November 2022, involving 636 cycles of in vitro fertilization/intracytoplasmic sperm injection (IVF/ICSI). Subgroup analyses were performed using cut-off values of 6.4 for free androgen index (FAI), 9.5 µmol/L for DHEAS. Pregnancy and neonatal outcomes were compared between groups. Restricted cubic spline (RCS) was used to identify significant cut-off values affecting pregnancy.

**Results:**

Higher FAI levels (> 6.4) were associated with decrease in clinical pregnancy rate (PR) (50.61% vs. 41.66%, *p* = 0.024), live birth rate (LBR) (42.42% vs. 32.35%, *p* = 0.011). When DHEAS levels exceeded 9.5 µmol/L, there was a significant decrease in clinical PR (51.27% vs. 42.73%, *P* = 0.039), LBR (42.73% vs. 32.73%, *P* = 0.012). Negative correlations were also observed between DHEAS levels and cumulative pregnancy rate (70.57% vs 56.62% *p* = 0.002) and cumulative live birth rate (CLBR) (59.35% vs 43.37%, *p* = 0.0007). Both FAI and DHEAS elevated is associated with the lowest clinical pregnancy rate (37.84%). Conversely, when solely FAI is elevated, the pregnancy rate increases to 52.38%, while an elevation in DHEAS alone is associated with a pregnancy rate of, both of which are lower than when neither FAI nor DHEAS are elevated (60.68%). The live birth rates exhibit a similar trend (30.00% vs 40.00% vs 41.83% vs 44.48%). RCS revealed a significant decrease in CPR and CLBR when DHEA levels exceeded 7.69 umol/L, while the cut-off value of FAI was 6.36 for CPR and CLBR.

**Conclusion:**

In conclusion, PCOS patients with biochemical hyperandrogenism show unsatisfactory clinical PR and CLBR when undergoing assisted reproductive technology (ART). This may be attributed to the influence of both adrenal-derived DHEAS and ovarian-derived FAI on the unfavorable pregnancy outcomes.

**Supplementary Information:**

The online version contains supplementary material available at 10.1186/s12958-024-01212-y.

## Introduction

Polycystic Ovary Syndrome (PCOS) has been labeled as the most prevalent endocrine disorder among women of reproductive age, contributing to a 3–10% infertility rate within this demographic. Moreover, PCOS is responsible for a considerable portion (30–60%) of ovulatory disorders associated with infertility [1, 2].

Hyperandrogenism is associated with the morphological manifestation of polycystic ovaries [3]. Current opinions suggest that androgens stimulate the recruitment of small follicles while suppressing the selection of dominant follicles, consequently leading to anovulation [4]. High levels of androgens have been established as fundamental clinical features and necessary biochemical diagnostic criteria for PCOS [5]. The Recommendations from the 2023 guideline for the assessment and management of PCOS suggests that the biochemical diagnosis of hyperandrogenism includes the measurement of serum total testosterone, free testosterone. The assessment of free testosterone can be achieved by calculating the free androgen index (FAI). In cases where testosterone or free testosterone levels are not elevated, it may be appropriate to consider testing for androstenedione and dehydroepiandrosterone sulfate (DHEAS) [6–8].

Testosterone and androstenedione are primarily derived from the ovaries which consist of the main source of androgens in PCOS [9]. while DHEA and DHEAS are mainly produced by the adrenal glands [10] Evidence suggests that patients with elevated baseline serum androgen levels (> 40 ng/dL) have a lower probability of achieving sustained pregnancies following assisted reproductive therapies. Elevated levels of free androgen index (FAI) (≥ 3.08) have been linked to a reduced clinical pregnancy rate in PCOS patients undergoing assisted reproductive techniques (ART) [11–15]. Approximately 20 to 30% of PCOS patients are demonstrated to have excessed adrenal androgen secretion, primarily indicated by elevated levels of DHEAS [16].Individuals with hyperandrogenism who have elevated DHEAS levels tend to exhibit a leaner physique, lower insulin levels, and a more favorable metabolic profile, indicating that elevated DHEAS levels could potentially confer a protective effect against metabolic syndrome [17]. Furthermore, a potential association exists between increased DHEAS levels in PCOS patients and an increased risk of miscarriage [17–19]. The reference range for biochemical hyperandrogenism may vary across different races and studies. American Society for Reproductive Medicine (ASRM) defined DHEA > 400 ng/dL and/or DHEAS > 350 µg/dL, free testosterone > 4.0 pg/mL, and total testosterone > 40 ng/dL as abnormal. Enrico’s study, on the other hand, considered serum testosterone > 55 ng/dL and/or serum DHEAS higher than 3 mcg/mL (> 7.8 mmol/L) as indicators of biochemical hyperandrogenism. However, in the East Asian population, the cutoff values for biochemical hyperandrogenism in PCOS patients are still under investigation. Based on these evidences, we hypothesize that DHEAS and FAI may influence pregnancy and neonatal outcomes in PCOS patients undergoing ART. This retrospective study seeks to investigate the impact of DHEAS and FAI on reproductive outcomes in women diagnosed with PCOS, with the objective of identifying the precise threshold values that significantly affect pregnancy outcomes. Consequently, this study provides valuable insights for pre-treatment interventions and the enhancement of reproductive outcomes.

## Method

### Study design and participants

This retrospective cohort study included 1647 PCOS patients who underwent in vitro fertilization/intracytoplasmic sperm injection (IVF/ICSI) cycles at the Reproductive Medicine center of the First Affiliated Hospital of Sun Yat-sen University from January 2015 to November 2022. The diagnosis of PCOS was based on the Rotterdam criteria [4]. Exclusion criteria included patients with congenital adrenal hyperplasia, androgen-secreting masses, oocyte donation cycles, and PGT cycles. Patients with canceled cycles, missing data on FAI, DHEAS, or those lost to follow-up were excluded. Eventually, 636 cycles were included in the analysis (Supplemental Fig. 1).The cut-off value of 6.4 for FAI is based on the FAI cutoff value for hyperandrogenemia in Chinese women [20] and a cut-off value of 9.5umol/l for DHEAS is then based on DHEAS outliers in the literature [8, 21]. A total of 883 embryo transfer cycles were included. This study has obtained approval from the Institutional Ethics Committee(「2023」759), and does not require informed consent. The study subjects mainly consisted of patients from Guangzhou city.

### COS stimulation and transfer protocol

Therapeutic strategies in this study followed established guidelines, which included ovarian stimulation, oocyte retrieval, and embryo transfers. Various controlled ovarian stimulation (COS) regimens were used. The choice of regimen was based on individual patient circumstances. Oocyte maturation was induced by administering hCG (6000 to 10,000 IU) when at least three follicles measured 17 mm or at least two follicles reached 18 mm in diameter. Transvaginal ultrasound-guided follicle aspiration was performed 35–36 h later to retrieve the oocytes, which were fertilized using either IVF or ICSI, depending on sperm quality. On the third day after oocyte retrieval, a maximum of two embryos were transferred. The quality of cleavage stage embryos was assessed using Cummins criteria [22].For frozen embryo transfer cycles, endometrium preparation protocols depended on the patient’s menstrual cycle, including natural and hormone replacement cycles. Progesterone preparation for luteal support began either three days before day-3 embryo transfer or five days before blastocyst transfer. HCG tests were conducted on day 14 after embryo transfer. If the result of HCG test was positive, luteal support would continue until 10 weeks of gestation. Ultrasound examination was performed 28 days after embryo transfer, and clinical pregnancy was diagnosed when gestational sac was detected. Live birth was defined as the presence of heartbeat, respiration, umbilical cord pulsation, and voluntary muscle movement at birth, encompassing both full-term and premature infants.

### Measurement of serum hormone

Venous blood samples were collected in standard blood collection tubes containing heparin (Vacutainer™, Beckton Dickinson, Franklin Lakes, NJ, USA) before the controlled ovarian stimulation cycle. DHEAS, testosterone, sex hormone-binding globuline (SHBG) were measured by using highly accurate tandem mass spectrometry(LC-MS/MS). FAI was calculated using the formula: [tT (nmol/L) ÷ SHBG (nmol/L)] × 100. There was no change in the assay during the study and the laboratory regularly performed calibration to minimize variation of the results associated with time and reagent batch renewal.

### Pregnancy and neonatal outcomes

We assessed several primary outcomes in our study, including clinical pregnancy rate (PR), live birth rate (LBR), cumulative pregnancy rate (CPR) and cumulative live birth rate (CLBR) per patient. CPR referred to pregnancy episodes in fresh and subsequent frozen-thawed embryo transfer cycles, while CLBR was defined as live birth episodes in fresh and subsequent frozen-thawed embryo transfer cycles. Additionally, secondary outcomes included caesarean deliveries, pregnancy loss, early miscarriages, late miscarriages, and intrapartum complications. Preterm birth was defined as a baby born before 37 weeks of gestation, while full-term birth referred to a baby born after 37 weeks and before 41 weeks of gestation. Pregnancy loss encompassed miscarriage, stillbirth and genetic terminations. Specifically, early miscarriage was defined as spontaneous abortion occurring before 12 weeks of gestation, while late miscarriage occurred between 12 weeks and 24 weeks of gestation. Neonatal outcomes included birth weight, birth length, macrosomia, and congenital malformations. Macrosomia weight was defined was the fetus being > 4 kg. Only major malformations were considered and recorded according to the International Classification of Diseases, tenth revision (ICD-10). Congenital malformations involved in this study included cleft palate, congenital heart disease (CHD), leg malformations, and chromosomal abnormalities. Discordant malformations were defined as a pair of twins with one affected and one unaffected fetus or twin pairs with different malformations. Neonatal outcomes involving congenital malformations were obtained from telephone follow-up.

### Statistical analysis

In this study, FAI and DHEAS were categorized as categorical and binary variables by means of cut-off points(FAI > 6.4,FAI < 6.4,DHEAS > 9.5umol/l, DHEAS < 9.5umol/l). Pregnancy and neonatal outcomes were examined among different groups. Data were collected using Excel software, and analyzed using SPSS 26.0 and R4.2.0 software. The measurement data were described by mean ± standard deviation, and the count data were described by frequency and constitutive ratio. If the quantitative data follows a normal distribution with variance equal to chi-square, paired t-test was used, while rank-sum test was used when the data does not follow a normal distribution. Qualitative data were tested using chi-square test or Fisher’s exact probability method. We explored the associations between FAI or DHEAS measurements as continuous variables and the primary outcome. Univariable logistic regression analyses were performed to assess the association of each of the predictive factors with pregnancy outcomes. The predictors included in the multivariable logistic regression were selected based on the result of univariable logistic regression analyses (*P* < 0.05). The backward procedure for variable selection was applied for the multivariable logistic regression model. Restricted cubic spline analysis was used to examine the cut-off value of FAI and DHEAS as a continuous variable. Potential nonlinearity was tested by using a likelihood ratio test comparing the model with only a linear term against the model with linear and cubic spline terms. Restricted cubic spline with best fit knots according to the AIC rule was used to flexibly model the potential nonlinear effects of FAI and DHEAS. Restricted cubic spline modeling was conducted with R, version 4.2.0.

## Result

### Characteristics of the PCOS patients and IVF/ICSI treatments

1647 women with PCOS underwent a total of 1858 IVF stimulation cycles throughout this study period, of whom 1222 were excluded because of missing data regarding FAI. The baseline clinical features at the time of the stimulation cycle of the remaining 636 cycles are described in Table 1.

Generally, the infertility types showed significant differences in infertility types between different FAI groups (*p = 0.014*) that the incidence of secondary infertility was higher in the FAI > 6.4 group (43.28% vs. 31.47%), while the incidence of primary infertility was lower in the FAI > 6.4 group (56.72% vs. 68.53%). BMI showed statistically significant differences between the FAI < 6.4 and FAI > 6.4 group (*p* < 0.001). Higher BMI was observed in the FAI > 6.4 (24.43 kg/m^2^). However, no statistically significant difference was present regarding women’s age, duration of infertility, baseline levels of basal FSH or basal LH. Statistically significant differences in rates of normal fertilization and high-quality blastocyst embryos were also observed, while no significant difference of the Fertilization rate and Blastocyst formation rate was found.

**Table 1:** Baseline characteristics of PCOS patients among different groups

	FAI < 6.4 (*n* = 502)	FAI > 6.4 (*n* = 134)	*P*	DHEA < 9.5 umol/l (*n* = 401)	DHEA > 9.5 umol/l (*n* = 166)	*P*
Age	29.56$\pm$3.50	30.10$\pm$3.56	0.113	29.85$\pm$3.48	29.29$\pm$3.67	0.086
Body mass index kg/m^2^	21.87(20.00,24.24)	24.43(22.47,26.59)	< 0.001	22.21(20.08,24.97)	22.70(20.91,24.98)	0.066
Duration of infertility (years)	3.00(2.00,5.00)	4.00(2.00,6.00)	0.053	3.00(2.00,5.00)	3.00(2.00,5.00)	0.510
Type of infertility			0.014			0.173
Primary infertility	344(68.53%)	76(56.72%)		273(68.08%)	103(62.05%)	
Secondary infertility	158(31.47%)	58(43.28%)		128(31.92%)	63(37.95%)	
Protocol			0.197			0.357
Long GnRH agonist protocol	70(13.94%)	11(8.21%)		43(10.72%)	25(15.06%)	
GnRH antagonist protocol	420(83.67%)	119(88.81%)		347(86.53%)	137(82.53%)	
others	12(2.39%)	4(2.99%)		11(2.74%)	4(2.41%)	
Basic FSH	5.00$\pm$1.28	4.95$\pm$1.21	0.691	5.04$\pm$1.21	4.83$\pm$1.27	0.058
Basic LH	6.62$\pm$4.04	7.24$\pm$7.19	0.196	6.85$\pm$5.37	6.47$\pm$3.82	0.404
Number of retrieved oocytes	20.00(13.00,26.25)	20.00(14.00,29.00)	0.526	19.00(13.00,26.00)	20.00(14.00,29.00)	0.134
Fertilization rate	0.81(0.67,0.90)	0.77(0.63,0.88)	0.097	0.81(0.67,0.90)	0.78(0.61,0.89)	0.096
High quality embryo rate	0.75(0.50,1.00)	0.76(0.34,1.00)	0.828	0.77(0.50,1.00)	0.74(0.50,1.00)	0.247

PCOS: polycystic ovary syndrome; FSH: follicle-stimulating hormone; LH: luteinizing hormone; FAI: free androgen index; DHEAS: dehydroepiandrosterone sulfate. *p* < 0.05 is considered to have significant statistical significance

### Reproductive outcomes in different phenotypes of PCOS patients

Pregnancy outcomes of PCOS patients separated by particular DHEAS or FAI cut-off value are presented in Table 2. A total of 883 embryo transfer cycles were included in this study. Our findings demonstrated that FAI > 6.4 was associated with a significant decrease in the clinical PR (50.61% vs. 41.66%, *p* = 0.024) and LBR (42.42% vs. 32.35%, *p* = 0.011). The early miscarriage rate (10.75% vs. 13.33%, *p* = 0.372) and late miscarriage rate (3.78% vs. 5.89%, *p* = 0.371) were increased, although these differences did not reach statistical significance. The rate of cesarean Sect. (65.86% vs. 70.15%) and the incidence of pregnancy complications (21.06% vs. 21.42%) were also investigated, revealing no significant differences between the two groups.

Similarly, we examined the influence of different DHEAS levels on pregnancy outcomes. Our results demonstrated a significant decrease in the clinical PR (51.27% vs. 42.73%, *p* = 0.039) and LBR (42.73% vs. 32.73%, *p* = 0.010) when DHEAS levels exceeded 9.5 µmol/L. Notably, the rate of late miscarriage exhibited a significant increase (1.77% vs. 8.51%, *p* = 0.004). Additionally, there was a trend towards an increased early miscarriage rate, but the difference was not statistically significant (14.13% vs. 13.90%, *p* = 0.866). No significant differences in rate of cesarean Sect. (70.29% vs. 60.27%) or the incidence of pregnancy complications (28.94% vs. 37.50%) were observed between the two groups. When DHEAS levels exceeded 9.5 µmol/L, both CPR (*p* = 0.002) and CLBR (*p* = 0.007) were significantly decreased.

**Table 2:** The reproductive outcomes in different phenotypes of PCOS patients

	FAI < 6.4 (*n* = 679)	FAI > 6.4 (*n* = 204)	*P*	DHEAS < 9.5 umol/l (*n* = 552)	DHEAS > 9.5 umol/l (*n* = 220)	*P*
Clinical pregnancy rate	50.61(344/679)	41.66(85/204)	0.024	51.27(283/552)	42.73(94/220)	0.039
Multiple pregnancies rate	14.12(42/344)	16.47(14/85)	0.300	11.31(32/283)	11.70(11/94)	1.000
Live birth rates	42.42(288/679)	32.35(66/204)	0.011	42.73(238/552)	32.73(72/220)	0.010
Abnormal pregnancy	3.20(11/344)	2.35(2/85)	0.684	3.18(9/283)	2.13(2/94)	0.738
Early miscarriage rate	10.75(43/344)	13.33(14/85)	0.372	14.13(40/283)	14.90(14/94)	0.866
Late miscarriage rate	3.78(13/344)	5.89(5/85)	0.371	1.77(5/283)	8.51(8/94)	0.004
Pregnancy Complications	21.06(60/288)	21.42(14/66	1.000	28.94(69/238)	37.50(27/72)	0.221
Cumulative pregnancy rate	68.53(344/502)	63.43(85/134)	0.311	70.57(283/401)	56.62(94/166)	0.002
Cumulative live birth rate	57.37(288/502)	49.25(66/134)	0.114	59.35(238/401)	43.37(72/166)	0.0007

PCOS: polycystic ovary syndrome; FAI: free androgen index; DHEAS: dehydroepiandrosterone sulfate. *p*<0.05 is considered to have significant statistical significance

We conducted further analysis on 772 cycles that had both DHEAS and FAI results to examine the influence of various levels of DHEAS and FAI on pregnancy outcomes.(Table 3) The findings of our study indicate that the concurrent elevation of both FAI and DHEAS is associated with the lowest clinical pregnancy rate (37.84%). Conversely, when only DHEAS is elevated, the pregnancy rate increases to 51.63%, while an elevation in FAI alone is associated with a higher pregnancy rate of 52.38%. The highest pregnancy rate was observed when neither FAI nor DHEAS was elevated(60.68%). The live birth rates exhibit a similar trend(30.00% vs. 40.00% vs. 41.83% vs. 51.36%). These results demonstrate a noteworthy association between elevated levels of both FAI and DHEAS and a substantial rise in the incidence of late miscarriage rate(7.14%). Moreover, when solely FAI is elevated, the late miscarriage rate is 5.45%, whereas an elevation in only DHEAS corresponds to a late miscarriage rate of 2.53%(Table 3).

**Table 3:** Various levels of DHEAS and FAI on pregnancy outcomes

	FAI < 6.4 DHEAS < 9.5 umol/l (*n* = 440)	FAI > 6.4 DHEAS > 9.5 umol/l (*n* = 74)	FAI > 6.4 DHEAS < 9.5 umol/l (*n* = 105)	FAI < 6.4 DHEAS > 9.5 umol/l (*n* = 153)	*P*
Clinical pregnancy rate	60.68(267/440)	37.84(28/74)	52.38(55/105)	51.63(79/153)	0.002
Multiple pregnancies rate	7.87(21/267)	17.86(5/28)	20.00(11/55)	11.39(9/79)	0.0328
Live birth rates	51.36(226/440)	30.00(22/74)	40.00(42/105)	41.83(64/153)	0.001
Abnormal pregnancy	13.86(37/267)	25.00 (7/28)	25.45(14/55)	20.25(16/79)	0.093
Early miscarriage rate	11.24(30/267)	14.29(4/28)	18.18(10/55)	16.46(13/79)	0.418
Late miscarriage rate	4.12(11/267)	7.14(2/28)	5.45(3/55)	2.53(2/79)	0.712
Pregnancy Complications	0	3.57(1/28)	5.45(3/55)	0	0.380
Cumulative pregnancy rate	80.91(267/330)	58.33(28/48)	75.34(55/73)	68.10(79/116)	0.001
Cumulative live birth rate	68.48(226/330)	45.83(22/48)	57.53(42/73)	55.17(64/116)	0.003

PCOS: polycystic ovary syndrome; FAI: free androgen index; DHEAS: dehydroepiandrosterone sulfate. *p* <0.05 is considered to have significant statistical significance

### Neonatal outcomes in different phenotypes of PCOS patients

The neonatal outcomes in different phenotypes of PCOS patients are presented in Table 4. There was no statistically significant difference in birth weight (*p* = 0.305;0.419), macrosomia rate (*p* = 0.167;1.000), or congenital malformations (cleft palate, congenital heart disease, leg malformations, chromosomal abnormalities) among different phenotypes of PCOS patients.

**Table 4:** Neonatal outcomes in different phenotypes of PCOS patients

	FAI < 6.4 (*n* = 679)	FAI > 6.4 (*n* = 204)	*P*	DHEAS < 9.5 umol/l (*n* = 552)	DHEAS > 9.5 umol/l (*n* = 220)	*P*
Fetal sex			0.883			0.906
Male%	49.65(143/288)	51.52(34/66)		49.16(117/238)	47.22(34/72)	
Female%	50.35(145/288)	48.48(32/66)		50.84(121/238)	52.78(38/72)	
Birth Weight(g)	2962.45	2931.45	0.305	2960.13	2960.73	0.419
Birth Length(cm)	48.48	48.26	0.908	48.35	48.33	0.350
Macrosomia rate	1.74(5/288)	4.55(3/66)	0.167	2.52(6/238)	2.78(2/72)	1.000
Congenital malformations	3.12(9/288)	0	0.374	2.94(7/238)	1.39(1/72)	0.686
Caesarean section delivery	65.86(191/288)	70.15(47/66)	0.566.	70.29(168/238)	60.27(44/72)	0.117
Pregnancy Complications	21.06(60/288)	21.42(3/14)	0.709	28.94(69/238)	37.50(27/72)	1.000

PCOS: polycystic ovary syndrome; FAI: free androgen index; DHEAS: dehydroepiandrosterone sulfate. *p* < 0.05 is considered to have significant statistical significance

### Turning point of the correlation between DHEAS, FAI and reproductive outcomes

In order to further determine the cutoff values of DHEAS and FAI that impact pregnancy outcomes, we employed a multivariate regression model(supplemental Table 1) and Restricted Cubic Spline (RCS). Our results revealed that age and BMI were significant factors influencing pregnancy outcomes. After adjusting for age and BMI, we observed a significant decrease in the clinical pregnancy rate when DHEAS exceeded 9.39 µmol/mL. Similarly, when DHEAS exceeded 7.69 µmol/mL, both the cumulative pregnancy rate and cumulative live birth rate showed a significant decrease. Furthermore, the cut-off value for FAI’s impact on the clinical PR was determined as 6.29, while the cutoff value for the CPR and CLBR was determined as 6.36.

**Fig. 1:**
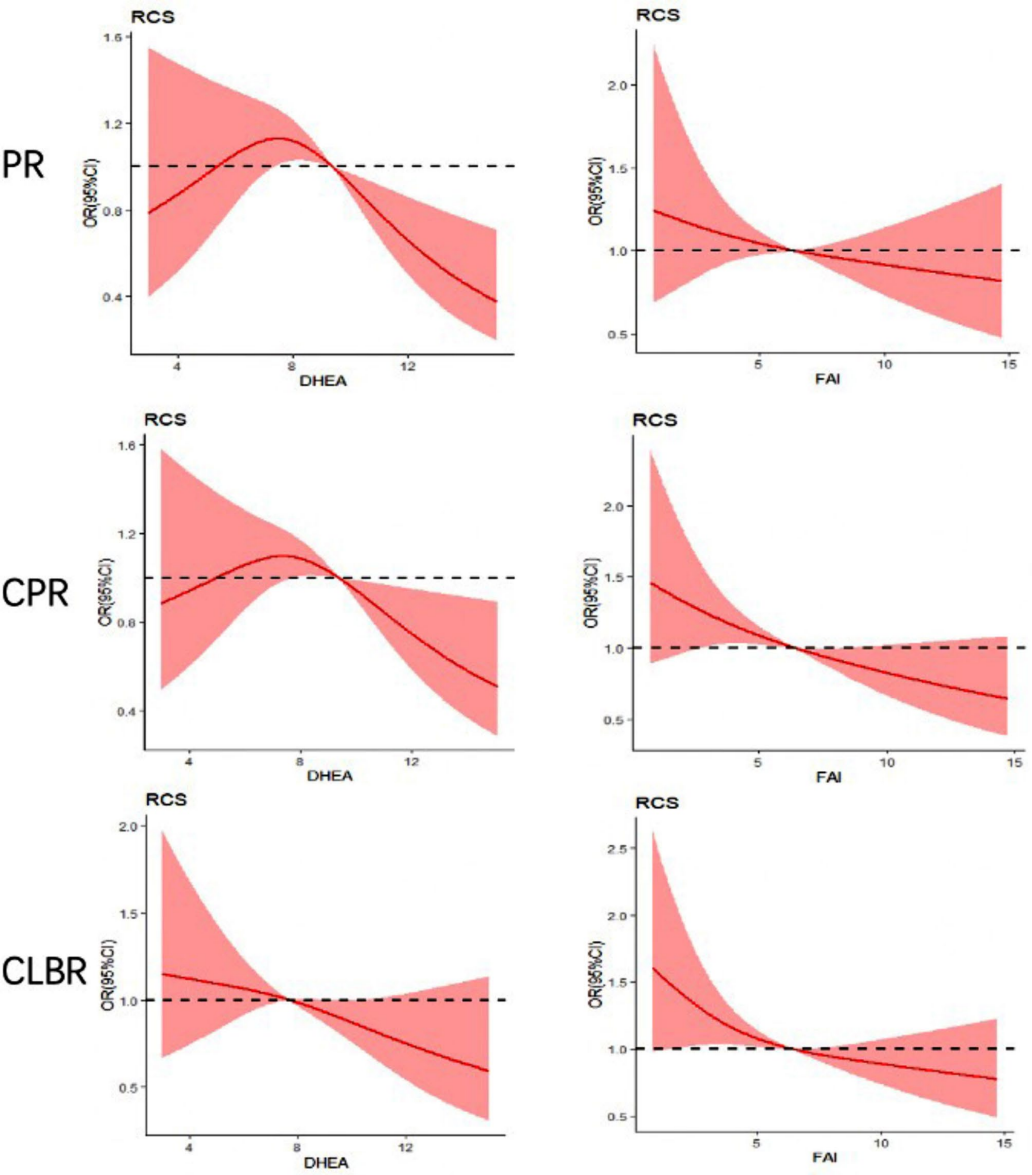
Restricted Cubic Spline for Turning point of DHEAS, FAI. PR: clinical pregnancy rate; CPR: cumulative pregnancy rate; CLBR: cumulative live birth rate; The intersection of the dashed line and the solid line is the turning point

## Discussion

This retrospective study suggests that both adrenal-derived DHEAS and ovarian-derived FAI, may contribute to poorer pregnancy outcomes in PCOS patients undergoing ART. By utilizing RCS analysis, our findings provide evidence that FAI levels ≥ 6.36 has a significantly negative impact on CPR and CLBR while cutoff value for DHEAS was determined as 7.69 umol/L.

PCOS is an endocrine disorder characterized by excessive androgens [23]. While ovarian hyperandrogenism is commonly associated with PCOS, the role of adrenal hyperandrogenism in clinical manifestations or hormonal profiles of PCOS requires further clarification [24–26]. The FAI is a percentage that represents the ratio of total testosterone to sex hormone-binding globulin (SHBG). It is utilized as a measure of the biologically active fraction of testosterone in circulation. Several studies have suggested that elevated levels of FAI may have negative implications for pregnancy outcomes in patients with PCOS. Zhang et al. found that FAI and free testosterone (FT) were significant factors associated with fertility outcomes in infertile PCOS patients, with FAI showing a significant correlation with ovulations per cycle, clinical pregnancy, hCG positive, and live birth rates. When FAI exceeded 7.85, the pregnancy rate and live birth rate significantly decreased, while the conception rate increased inversely [15, 27], FAI may serve as a significant predictor for conception, pregnancy, and LBR [28, 29]. Additionally, Kuang et al. demonstrated that combining FAI with factors such as age and insulin levels could predict at least one pregnancy outcome [30, 31]. In a similar vein, our findings suggest a potential association between elevated FAI levels and reduced clinical PR, CPR, and LBR. This correlation may be attributed to the increased levels of insulin resistance, which could be linked to free androgen and play a critical role in the preparation of the endometrium for embryo implantation [5, 32]. However, the abnormal values of FAI in the Asian population are still under investigation. Our study demonstrates that FAI levels ≥ 6.36 have a detrimental effect on CPR and CLBR.

Approximately 20 to 30% of PCOS patients demonstrate adrenal androgen excess, primarily indicated by elevated levels of DHEAS [16]. Previous studies have indicated that PCOS patients with elevated DHEAS levels exhibit higher values of testosterone (T) and androstenedione (A) against those with normal DHEAS levels, potentially indicating a link between abnormal DHEAS levels to metabolic dysfunctions [24]. Additionally, among patients with diminished ovarian reserve, DHEAS concentrations showed a significant negative correlation with the number of follicles on the day of oocyte maturation triggering [33–35]. However, studies that explored the association between DHEAS levels and PR or LBR in PCOS patients are still limited. Our study provided evidences that increased DHEAS levels may be correlated with pregnancy outcomes, including PR, CPR, and CLBR. Notably, when DHEAS levels were ≥ 7.69umol/ml, there was a significant negative impact on the CPR and CLBR. Similarly, a separate study indicated that DHEA and DHEAS could serve as better predictors of abnormal anthropometric and biochemical parameters in women with PCOS, which may be associated with fertility issues [36, 37].

According to the presented results, it is evident that the concurrent elevation of both FAI and DHEAS is associated with the lowest pregnancy rate and live birth rate. Conversely, when only FAI is elevated while DHEAS remains unaltered, both the pregnancy rate and live birth rate are comparatively lower than those with solely elevated DHEAS levels. This result implies that androgens originating from the ovaries exhibit a more pronounced influence on infertility in individuals with PCOS when compared with androgens, particularly DHEAS, derived from the adrenal glands. According to the recommendations for the assessment and management of PCOS, it is advised to include the measurement of serum total testosterone and free testosterone in the biochemical diagnosis of hyperandrogenism. In instances where testosterone or free testosterone levels are not elevated, it may be deemed appropriate to consider conducting tests for androstenedione and dehydroepiandrosterone sulfate (DHEAS) [6]. The results of our study also support this suggestion.

The reference range for biochemical hyperandrogenism may vary across different laboratories and studies. Studies such as American Society for Reproductive Medicine (ASRM) and Enrico’s study have defined abnormal levels differently. ARSM defined DHEA > 400 ng/dL and/or DHEAS > 350 µg/dL, free testosterone > 4.0 pg/mL, and total testosterone > 40 ng/dL as abnormal. Enrico’s study, on the other hand, considered serum testosterone > 55 ng/dL and/or serum DHEAS higher than 3 mg/mL (> 7.8 mmol/L) as indicators of biochemical hyperandrogenism [24]. It is important to note that these studies primarily focused on populations of European descent, and more research is needed to investigate these associations in Asian populations. In our study, we found that DHEAS levels exceeding 9.5 umol/L or FAI levels exceeding 6.4 were associated with decreased CPR and CLBR. To determine the precise cutoff values for DHEAS and FAI, we conducted RCS analysis. Age and BMI were identified as relevant factors influencing pregnancy outcomes, and they were included in the regression analysis. Ultimately, our analysis revealed a significant decrease in CPR and CLBR when DHEAS levels exceeded 7.69 umol/L and when FAI levels were ≥ 6.36. Given that the ASRM considers DHEAS levels above 9.4 umol/L as abnormal, we suggest that a threshold of 7.69 umol/L for DHEAS may be more indicative of decreased CPR and CLBR in PCOS patients. These findings have important implications for clinical management. Further research is needed to corroborate these results and explore their applicability in different populations.

Currently, the primary treatment options for PCOS include oral contraceptives that suppress testosterone levels and medications such as metformin, inositol, GLP-1 receptor agonists (GLP-1RA), and sodium-glucose cotransporter-2 (SGLT-2i) inhibitors that enhance insulin sensitivity. Combined oral contraceptive pills (COCP) effectively decrease LH production, thereby inhibiting steroidogenesis in the ovaries and increasing the production of SHBG, resulting in reduced free testosterone levels and an anti-androgenic effect [38]. However, further research is required to determine the optimal timing for intervention in PCOS patients with elevated androgen levels. The findings of the presented study indicate that a significant decline in the cumulative pregnancy rate among patients occurs when DHEAS levels exceed 7.69umol/l and FAI levels surpass 6.36, thus suggesting the potential necessity of treatment for elevated androgen levels in PCOS when DHEAS exceeds 7.69umol/l and FAI is higher than 6.36.

There are several limitations to this study. Firstly, the data used to develop the model were obtained from a single center with a relatively small sample size. Further multi-center randomized controlled trials with larger sample sizes are warranted to further validate the findings. Secondly, the patients included in this study were predominantly of slim physique with a lower proportion of obesity. Consequently, the generalizability of the cutoff value to obese populations may yield less optimal outcomes.

## Conclusions

In conclusion, elevated levels of androgens, such as DHEAS from adrenal sources or FAI from ovarian sources, are associated with reduced CPR and LBR in patients with PCOS undergoing ART. These findings emphasize that hyperandrogenism contributes to poorer pregnancy outcomes. It is crucial to acknowledge that though free androgens exhibit a more pronounced effect, the significance of dehydroepiandrosterone sulfate (DHEAS) should not be disregarded. Tailored interventions for managing hyperandrogenism in PCOS patients undergoing ART are warranted.

### Electronic supplementary material

Below is the link to the electronic supplementary material.

Supplementary Material 1

Supplementary Material 2

## Data Availability

The datasets generated and/or analyzed during the current study are not publicly available.
