# Supplementary material for: Impact of dehydroepiandrosterone sulfate and free androgen index on pregnancy and neonatal outcomes in PCOS patients
Source: Reprod Biol Endocrinol. 2024 Apr 16;22:43. doi: 10.1186/s12958-024-01212-y (PMC11020179; doi:10.1186/s12958-024-01212-y)
Supplement: Supplementary file 1 — Supplementary Material 1 [file 12958_2024_1212_MOESM1_ESM.docx]

Supplemental table 1 Multivariate logistic regression analysis for clinical pregnancy rate

|  | B | *P* | 95%CI |
| --- | --- | --- | --- |
| Age | -0.091 | 0.033 | -0.023~-0.001 |
| BMI | -0.046 | 0.010 | -0.023~-0.003 |
| DHEAS | -0.089 | 0.035 | -0.027~-0.001 |
| FAI | -0.106 | 0.022 | -0.023~-0.002 |
