# Supplementary figures and images for: Impact of dehydroepiandrosterone sulfate and free androgen index on pregnancy and neonatal outcomes in PCOS patients
Source: Reprod Biol Endocrinol. 2024 Apr 16;22:43. doi: 10.1186/s12958-024-01212-y (PMC11020179; doi:10.1186/s12958-024-01212-y)

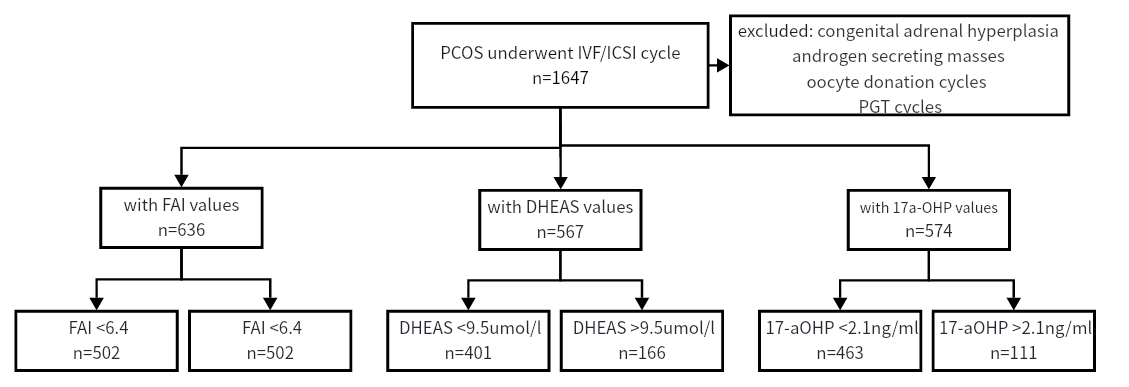

Supplement: Supplementary file 2 — Supplementary Material 2 [file 12958_2024_1212_MOESM2_ESM.tif]
